# Exploratory, Participatory and Iterative Assessment of Value: A Response to Recent Commentaries

**DOI:** 10.34172/ijhpm.2020.76

**Published:** 2020-05-20

**Authors:** Janneke P.C. Grutters, Tim M. Govers, Jorte Nijboer, Marcia Tummers, Gert Jan van der Wilt, Maroeska M. Rovers

**Affiliations:** ^1^Department for Health Evidence, Radboud Institute for Health Sciences, Radboud University Medical Center, Nijmegen, The Netherlands.; ^2^Department for Operating Rooms, Radboud Institute for Health Sciences, Radboud University Medical Center, Nijmegen, The Netherlands.; ^3^Radboud Institute for Health Sciences, Radboud University Medical Center, Nijmegen, The Netherlands.; ^4^Department for Health Evidence, Donders Institute for Brain, Cognition and Behaviour, Radboud University Medical Center, Nijmegen, The Netherlands.

We were deeply honoured that so many reputable scholars read our article and took the effort to write no less than eight thoughtful and interesting commentaries.^1-8^ Writing the original paper was sometimes a struggle, because it was an unconventional paper. However, we felt a strong urge to make these assessments – that are often confidential and not publicly available – public, and share our experiences to advance this important and timely field of research. We hoped that sharing our experiences would fuel the discussions on early health economic modeling, but these eight commentaries exceeded our expectations. We are grateful to the many suggestions for further improvement that were provided, and generally agree with all of them. Many interesting topics were raised that need further attention, such as the use of early health economic modeling within the context of early dialogues with payers and health technology assessment bodies at early stages of product development,^1,2^ the use of real world evidence,^3,8^ the complexity of interventions and systems,^6^ and model quality.^7^

In this response, we take the opportunity to further discuss three topics that were mentioned by multiple scholars: the dealing with uncertainty in early health economic modeling, the notion of value and the iterative nature of health economic modelling.

## Dealing With Uncertainty

In the assessments, we did not address parameter uncertainty by means of probabilistic sensitivity analysis (PSA), despite this being recommended as best practice in health economic modeling.^9^ As explained in the discussion section, we agree that it is informative to use PSA for uncertainties that are due to imprecision. If there is some (pilot) evidence on the effectiveness of the innovative technology, PSA is indeed recommended. A major advantage is that it allows for a value of information analysis, as suggested by Drummond.^1^ However, for an audience of non-experts in health economic modeling, these analyses require careful interpretation and explanation, as they can easily be misunderstood. The use of PSA is, in our view, more problematic in case there is not yet any evidence of effectiveness of the technology, which was the case for most of the technologies that were included in our study. They were not yet used in clinical practice, not even in pilot settings.

Several authors provide valuable suggestions for dealing with the lack of evidence in the early stages of development of a medical technology, such as clinical trial simulation and expert elicitation.^2,6^ Most of these focus on quantifying the known unknowns, with a focus on prediction. While in these cases PSA and value of information are technically possible, we argue that in such early stages exploration may be more valuable than prediction. For example, the use of expert elicitation may result in pseudo certainty if experts do not believe the innovation to be effective. Does this mean that we are certain that the innovation is not effective, or not cost-effective, and that there is no value in performing further research? Whether explorative or predictive analyses are more valuable may also depend on the public, and the aim of the assessment. For a technology developer, it may be more informative to learn under *what circumstances* the technology will be cost-effective, than learning *how likely* it is that the technology will be cost-effective.

In other fields of research, exploratory modeling is an important tool, which might be very useful in early health economic modeling, too. Exploratory modeling aims at providing decision support “even in the face of many irreducible uncertainties, by systematically exploring the consequences of a plethora of uncertainties – ranging from parametric uncertainties (eg, parameters ranges), over structural uncertainties (eg, different structures and models), to method uncertainties (eg, different modeling methods) – using computational models as scenario generators.”^10^ It stems from scenario thinking, but instead of calculating a pre-determined set of scenarios, in exploratory modeling a comprehensive set of all possible scenarios that can be envisaged is simulated. It might be viewed as an *n*-way sensitivity analysis, where *n* includes all uncertain parameters at all possible (instead of probable) values. Then, the researcher can analyze under which circumstances a technology or strategy is disputable (eg, not cost-effective), and under which circumstances it is most valuable. Thus, exploratory modeling involves searching through the set of outcomes using (many-objective) optimization algorithms.^11^ This also provides important information on what evidence should be collected, and what (adaptive) approach should be taken, for example in terms of coverage with evidence development. Kim et al refer to the use of early health economic modeling in ipilimumab for advanced melanoma in Australia.^3^ In the Netherlands, conditional reimbursement schemes with evidence collection are hardly based on uncertainties other than imprecision.^12^ With use of exploratory modeling, one could explore under which circumstances (eg, at which survival rate, or in case of which parallel developments in the control of the relevant disease condition) ipilimumab is cost-effective. An adaptive strategy can then be implemented where evidence is collected and clear thresholds are set: if a certain threshold (eg, survival rate) is reached, reimbursement of the drug can be continued, if not, reimbursement should be withdrawn. With such an exploratory approach, we completely agree with Kim et al that “early health economic modelling could bridge this gap by better conceptualising the risks and uncertainties for both payers and sponsors.”^3^ This does, however, require a broader notion of uncertainty than imprecision.^13,14^

## The Notion of Value

Many of the commentaries rightfully discussed our strong focus on cost-effectiveness. Of course, as we mentioned in the original paper, value of an innovative technology is much more than cost-effectiveness alone. We fully agree that “cost-effectiveness alone may be too reductive if taken as the only decision rule, and it would benefit from being used within a broader evaluation framework.”^2^ Let us clarify that this mainly resulted from the focus of the paper, which was to study whether early health economic modeling could distinguish ‘valuable’ from ‘non-valuable’ innovations.

In the underlying assessments we did not restrict our early analyses to economic modeling, but we did focus on the health economic part in this study. In the underlying advisory reports that were shared with the commissioners after the assessment, we did – qualitatively – include other aspects that might impact overall value, as we mentioned in the discussion section of our paper. Our assessments were technology-driven, which means that we assessed the technology and not the problem that the technology aimed to solve. We fully agree with Lehoux and Silva when they state that it is important to raise questions about the kinds of health innovations our systems of innovation should deliver.^4^ Otherwise, as a society we risk developing brilliant solutions for non-existing or relatively marginal problems.^15^ But also when an assessment is technology-driven, it is important to explore, already in the earliest stages of development, whether the intended innovation is deemed valuable and relevant by different stakeholders, and what possible barriers and facilitators could be for its use. As Teljeur and Ryan state, “giving them consideration at an early stage may provide an opportunity to tackle any issues that might otherwise only become apparent at a late stage of product development and hamper reimbursement and or uptake.”^7^

## The Iterative Nature of Health Economic Modelling

Our analysis was based on 32 assessments of 30 unique innovations. This means that while for two innovations we performed two consecutive assessments at different time points, the others were single assessments. We agree with Drummond that ideally “early stage health economic modeling is not a ‘one-time’ activity, but should be continuous and iterative, with the modeling being updated as more information becomes available, either about the technology itself or the environment in which it would be used.”^1,16^ This relates to the exploratory modeling and adaptive approach mentioned before, where new pieces of evidence can be integrated in the model and may warrant a change of strategy. Then indeed “the more relevant decision focus may be a ‘not yet’ or ‘yes, but (with conditions)’ decision, using economic modelling as an iterative and ongoing process.”^6^

We agree with Partington and Karnon that ideally such an iterative approach should include participatory methods that enable nuanced deliberation between stakeholders.^6^ A participatory approach to modeling could enhance the confidence stakeholders have in models, their relevance to the decision-makers and their account of uncertainty.^17^ Also, it would facilitate the broader notion of value as described in the previous paragraph, because stakeholders can deliberate on the value of the technology. To enable the iterative use of health economic models, not for just one technology but for different technologies aimed at a specific care pathway, Zawadzki and Hay suggest to make health economic models publicly available, “ideally in a standardized format to ensure consistent and complete representation of features, code, data sources, results, validation exercises, and policy recommendations.”^8^ This would indeed make health economic modeling more efficient, more transparent and will probably increase the quality of model-based assessments.^18^ However, it should be noted that the 32 assessments in our database were almost all commissioned by the developer of the technology, often a medtech company. Making the models and accompanying documentation publicly available may be in conflict with the confidentiality that is generally required in such early stages.

## Concluding Remarks

The commentaries seem to imply renewed interest in applying and further advancing the methodology of early health economic modeling. Our data were based on the first 32 assessments, and may capture an evolving process and learning curve that may not be representative of a steady state process.^7^ We therefore strongly encourage others to share their results, so that we can learn from each other and improve our assessments.

While it is informative for developers, early health economic modeling has the ability to have broader value, in bringing together the views of different stakeholders, and bridging the gap from the first idea to patient access. Ideally, it is an iterative and ongoing process, guiding the development, research and implementation of innovations to maximize its value for society. To achieve such as role, we do agree with Lehoux and Silva that we need to “transform our scholarly traditions.”^4^ In our opinion this is not restricted to broadening our notion of value, but also includes for example our definition and assessment of uncertainty. In this, early health economic modeling can learn from other disciplines that provide decision support. However, when transforming traditions and methodology, we always need to ensure that the assessments match the needs of the decision-makers they inform. We believe that iterative, participatory and exploratory modeling of value has the ability to enhance responsible and sustainable innovation.

## Ethical issues

Not applicable.

## Competing interests

Authors declare that they have no competing interests.

## Authors’ contributions

All coauthors have made a substantial contribution to the manuscript; they revised it critically for important content and approved the final version.

## Authors’ affiliations

^1^Department for Health Evidence, Radboud Institute for Health Sciences, Radboud University Medical Center, Nijmegen, The Netherlands. ^2^Department for Operating Rooms, Radboud Institute for Health Sciences, Radboud University Medical Center, Nijmegen, The Netherlands. ^3^Radboud Institute for Health Sciences, Radboud University Medical Center, Nijmegen, The Netherlands. ^4^Department for Health Evidence, Donders Institute for Brain, Cognition and Behaviour, Radboud University Medical Center, Nijmegen, The Netherlands.
